# Cross-cultural adaptation: South African Gestational Diabetes Mellitus Knowledge Questionnaire

**DOI:** 10.4102/safp.v67i1.5826

**Published:** 2025-01-13

**Authors:** Lorisha Manas, Tawanda Chivese, Ankia Coetzee, Magda Conradie, Linzette D. Morris

**Affiliations:** 1 Division of Epidemiology and Biostatistics, Department of Global Health, Faculty of Medicine and Health Sciences, Stellenbosch University, Cape Town, South Africa; 2 Department of Population Medicine, College of Medicine, QU Health, Qatar University, Doha, Qatar; 3 Division of Endocrinology, Department of Medicine, Faculty of Medicine and Health Sciences, Stellenbosch University, Cape Town, South Africa; 4 Department of Rehabilitation Sciences, College of Health Sciences, QU Health, Qatar University, Doha, Qatar

**Keywords:** gestational diabetes mellitus, knowledge assessment, health education, cross-cultural adaptation, South Africa, validation of questionnaire, validated questionnaire

## Abstract

**Background:**

Many serious adverse events associated with gestational diabetes mellitus (GDM) can be mitigated by timely glucose control during pregnancy, achieved through education and lifestyle choices. The aim of this study was to translate and cross-culturally adapt and test the preliminary internal consistency and test-retest reliability of the South African English, Afrikaans and isiXhosa versions of the GDM Knowledge Questionnaire (GDMKQ).

**Methods:**

A prospective, observational study was conducted at a high-risk antenatal clinic in South Africa. Pregnant women ≥ 18 years with GDM were consecutively sampled. Semantic equivalence between the original and adapted versions was assessed. Face and content validity, internal consistency and test–retest reliability were evaluated.

**Results:**

The three SA-GDMKQ versions demonstrated good face and content validity. For internal consistency, Cronbach’s alpha values were 0.534 for the Afrikaans version, 0.434 for the English version and 0.621 for the isiXhosa version. Test–retest reliability found kappa (standard error [s.e.]) values ranged between −0.03 (0.18) and 0.89 (0.13) for the English version, between −0.07 (0.18) and 0.53 (0.13) for the Afrikaans version and between 0.28 (0.18) and 0.87 (0.17) for the isiXhosa version. All versions of the SA-GDMKQ had a statistically significant (*p* < 0.001) positive linear correlation between the total scores.

**Conclusion:**

The English, Afrikaans and isiXhosa SA-GDMKQ versions were found to be feasible and easy to comprehend, although lower internal consistency and test–retest reliability were displayed. Further validation of the psychometric properties of the English, Afrikaans and isiXhosa versions of the SA-GDMKQ among larger sample groups is however warranted.

**Contribution:**

This study adds to the knowledge around developing and using culturally appropriate questionnaires and outcome measures in research and clinical practice.

## Introduction

Gestational diabetes mellitus (GDM), defined as ‘any degree of glucose intolerance identified at onset or first recognised during pregnancy’, is a major global public health concern.^1^ Although the true prevalence of GDM remains unknown because of inconsistent screening methods and diagnostic criteria,^2^ the International Diabetes Federation estimated that the global prevalence of hyperglycaemia in pregnancy is approximately 21.3 million (16.2%).^3^ Of these, the majority of cases (86.4%) were attributed to GDM.^3,4,5^ In low- and middle-income countries where there are limited resources and other socioeconomical and environmental factors that negatively affect health and access to healthcare, the projected burden of GDM is believed to be increased^6^ and under-managed. In South Africa, for instance, a lower-resource and diverse country comprising of a large proportion of populations living in rural and/or remote areas,^7^ the prevalence of GDM was estimated to range between 9.1% and 25.8%.^8,9^

If not managed properly, however, any degree of hyperglycaemia first detected in pregnancy, including GDM, is associated with a myriad of complications during and after pregnancy.^10,11^ Epidemiological surveillance indicates that GDM mothers and their offspring develop chronic non-communicable diseases (NCDs) easily.^10,11,12^ Gestational diabetes mellitus is specifically associated with increased risks of caesarian section, pre-eclampsia, gestational hypertension and type 2 diabetes post-partum for the mother.^10,11,12^ For the child, there are risks associated with neonatal hypoglycaemia, macrosomia and developing type 2 diabetes later in life.^10,11,12^

Many serious adverse events associated with GDM can however be mitigated through educating individuals about self-managing their condition and modifying their lifestyle choices.^13,14,15^ A good comprehension of GDM, adequate lifestyle modification and accurate self-management of their condition have been shown to translate into better glycaemic control, which may lead to reduced peri-natal, post-natal and long-term health complications for both mother and child.^13,14,15^ In primary health care settings, GDM patients are typically screened, monitored and managed by primary healthcare nurses and other health professionals. Part of the management of patients with GDM involves the implementation of health education strategies around self-monitoring and self-management of the condition.^13,14,15^ In areas where regular access to healthcare is, however, limited, for example, for patients living in remote or rural areas,^7^ these health educational strategies around self-management of the condition between their healthcare visits become even more vital. Educating patients about self-management is, however, applicable not only for patients in South Africa who live in rural or remote areas but also for patients in other countries where access to healthcare is limited because of similar factors.

To apply the most appropriate patient health education strategies and prescribe the most optimal self-management programme, the health professional needs to assess the patient’s baseline understanding of the condition and health literacy.^16^ At the time of the study, the authors were aware of only one questionnaire that assessed the knowledge base of patients around GDM, known as the Gestational Diabetes Mellitus Knowledge Questionnaire (GDMKQ). The original GDMKQ was developed in Malaysia by Hussain et al.^17^ and combined a number of existing questionnaires to produce a 15-item questionnaire. To our knowledge, however, no similar validated questionnaire fit for purpose in South Africa exists. This said, directly applying an already existing outcome measure that was developed for a different population and culture is not recommended.^18^ The use of cultural- and linguistic-inappropriate questionnaires may lead to misinterpretations of questions, inadequate application of the scoring system and anchores and/or references that are culturally not appropriate, which in turn may result in inaccurate measurement.^18^ This study therefore aimed to firstly translate the original GDMKQ to Afrikaans and isiXhosa and cross-culturally adapt the English, Afrikaans and isiXhosa versions for a South African population. Secondly, it aimed to preliminarily test the internal consistency and test–retest reliability of the English, Afrikaans and isiXhosa versions of the South African GDMKQ (SA-GDMKQ). The simultaneous translation of the GDMKQ into Afrikaans and isiXhosa languages and the adaptation of the English, Afrikaans and isiXhosa versions of the SA-GDMKQ are based on the fact that South Africa has 11 official languages, of which Afrikaans, English and isiXhosa are the most prevalent languages spoken in the western parts of the country.^19^

## Research methods and design

A prospective, observational study was conducted between February 2019 and October 2019, at a high-risk antenatal clinic situated in the Western Cape province of South Africa. The high-risk antenatal clinic is situated at South Africa’s second-largest public teaching hospital, which mainly serves patients from lower-income households from the surrounding and broader Western Cape province areas.^20^ Many of the patients who attend the clinic are from rural and/or remote areas within the catchment area of the hospital. An initial total sample size of 86 participants was calculated based on the intraclass correlation coefficient (ICC), assuming a significance level of 0.05 and power of 70%.^21^

**FIGURE 1:**
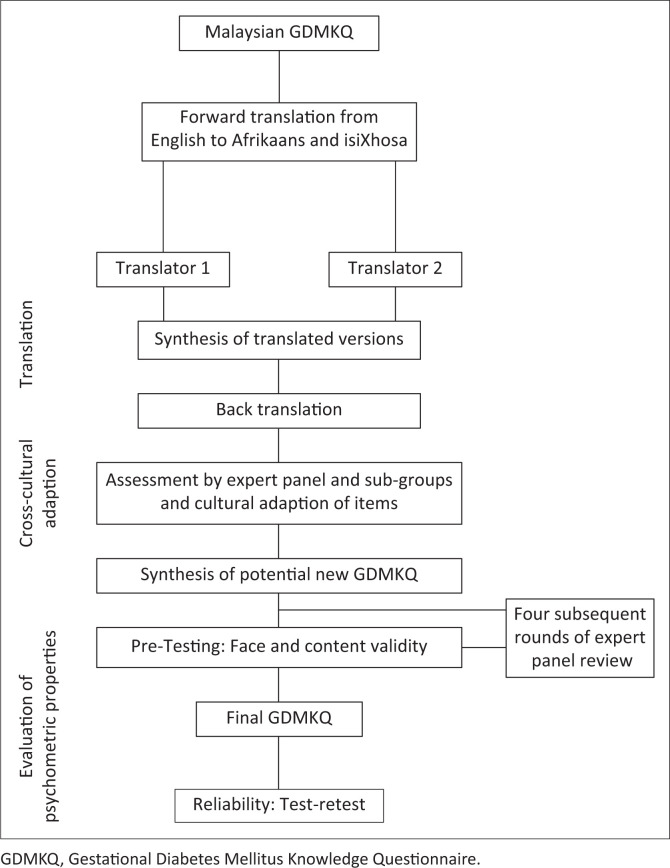
Illustration of study phases and study flow.

Participants were consecutively recruited and stratified into three language groups, namely, English, Afrikaans and isiXhosa. The inclusion criteria were as follows: pregnant participants clinically diagnosed with GDM according to the National Institute for Health and Care Excellence criteria (fasting glucose above 5.5 mmol/L and OGTT 2-h glucose over 7.8 mmol/L)^22^; participants who were able to read at a basic level in either English, Afrikaans or isiXhosa; participants who were > 18 years; and participants who attended the high-risk antenatal clinic during the study period. Female patients attending the clinic who were unable to comprehend what was expected of them if they participated in the study were excluded from the study. The study was conducted during the operating hours of the clinic, and there was no interference with the daily operations of the clinic. On enrolment into the study, participants were provided with an informed consent form and a socio-demographic questionnaire aimed at extracting the following data: age, education level, living area, number of pregnancies, duration of pregnancy, language, ethnicity, employment status, among others. These documents were provided in the preferred language of the participant. The study consisted of three phases. Phase 1 involved the translation and cross-cultural adaptation of the GDMKQ, phase 2 involved the preliminary testing of the newly adapted questionnaires’ face and content validation, and phase 3 involved the evaluation of psychometric properties of the newly adapted questionnaires in terms of internal consistency and test–retest validation. Figure 1 illustrates the study phases and study flow.

### Phase 1: Translation and cross-cultural adaptation

The original English version of the GDMKQ consists of 15 multiple-choice questions.^17^ The choices ‘a–c’ provide three possible answers from which the patient can choose, one of which is the absolute correct answer. Choice ‘d’ is the ‘I do not know’ option and is present on all 15 questions of the GDMKQ. The questionnaire consists of the following domains: *basic GDM knowledge* (questions 1–3), *risk factors* (questions 4–6), *diet and food management* (questions 7–9), *treatment and management* (questions 10–12) and *complications and outcomes* (questions 13–15).^17^ The answers provided are graded against an answer sheet that contains the correct answer for each question. For each correct answer, one mark is allocated, and for an incorrect answer or ‘I do not know’ option, a score of 0 is allocated. Higher scores indicate better knowledge about GDM.^17^ Permission to use the original English GDMKQ was obtained from the development authors.

The Strengthening the Reporting of Observational Studies in Epidemiology (STROBE) guideline for observational studies was followed to report this study.^23^ The translation and the cross-cultural adaptation process followed in this study were based on guidelines outlined by Beaton et al.^24^ Because of the unique context of South Africa, however, a slightly different approach was used. The original GDMKQ was translated into two languages simultaneously, and the three versions, namely, the English, Afrikaans and isiXhosa versions, of the GDMKQ were simultaneously cross-culturally adapted. A step-wise process in the translation of the two versions and the cross-cultural adaptation of the three versions of the GDMKQ was generally followed^25^: Step 1 – forward translation of original English version of the GDMKQ to Afrikaans and isiXhosa; Step 2 – synthesis of translations; Step 3 – backward translation; Step 4 – assessment by expert panel and cultural adaptation of items of the English, Afrikaans and isiXhosa versions of the GDMKQ; and Step 5 – face and content validation of the three versions of the SA-GDMKQ by participant subgroups.

Forward and back translation of the original English GDMKQ to Afrikaans and isiXhosa was conducted by professional and independent freelance translators, not informed about the project details. For the cross-cultural adaptation process, an expert committee was identified and invited (via email) to participate in the study. The expert committee included two endocrinologists, two obstetricians and gynaecologists, three dieticians, one biostatistician and one diabetes educator, which was aligned with guidelines stipulated for the compilation of the expert panel by Stewart et al.^18^ Three sub-groups of participants (*n* = 30, with *n* = 10 per language group) were randomly selected per stratum to evaluate semantic and conceptual equivalence in relation to the original items of the translated versions of the GDMKQ. To improve applicability, the expert panel and participants were asked to review the Malaysian GDMKQ (M-GDMKQ) in order to identify factors that were not appropriate to the context and suggest changes towards cross-cultural applicability. The principle investigator coded data obtained during the cross-cultural adaptation process in common themes and analysed by identifying key themes. The principal investigator collated all the changes and sent them to the expert panel until a consensus was reached and no more changes were recommended. Multiple rounds were expected to be conducted until a consensus was reached.

### Phase 2: Pre-testing – Face and content validation

To determine face and content validity, another three participant sub-groups (*n* = 30, with *n* = 10 per language group) were randomly selected to participate in the pre-testing phase of the study. Face and content validity was assessed qualitatively.^26^ For each participant, time was kept using a calibrated stopwatch, and the data were recorded. The sub-groups then completed a questionnaire relating to the ease of completing, acceptability, applicability and comprehensibility of the cross-culturally adapted and translated SA-GDMKQ versions. The sub-groups were asked to also provide any suggestions as to how the questionnaire should be further modified. The subgroup’s participants were included in the group of participants who participated in phase 3.

### Phase 3: Internal consistency and test–retest reliability testing

Internal consistency was investigated in questionnaires without missing data. To ascertain test–retest reliability, the participants were asked to complete the SA-GDMKQ twice, in their home language. The period between the completion of the two questionnaires was 2 weeks, at the next outpatient appointment. The participants completed the SA-GDMKQ individually at the high-risk antenatal clinic, in an isolated room, at both time points.

### Statistical analysis

Analyses were performed using STATA 15.1 (for Windows) and Statistical Package for the Social Sciences (SPSS) (IBM). Mean and standard deviation (s.d.) were reported for all normally distributed variables, and median and interquartile range (IQR) were reported for variables that were not normally distributed. Categorical variables were reported by frequencies and proportions. To compare variables, a one-way analysis of variance (ANOVA) hypothesis test was conducted for data that were normally distributed and a Kruskal–Wallis test for data that were not normally distributed. Chi-squared or Fisher’s exact test was used for categorical data. For the other quantitative data from the questionnaire (yes/no responses), frequencies and percentages were presented. Psychometric testing included face and content validity, internal consistency (Cronbach’s alpha – α) and test–retest reliability (Spearman’s correlation and Cohen’s kappa). A Cronbach’s α of 0.7 was considered acceptable, with 0.8 reflecting excellent internal consistency.^27^ Test–retest reliability measures reproducibility and was quantified by Spearman’s rho correlations coefficient (total score) and Cohen’s kappa (per question).^28^ A Spearman’s rho of > 0.6 was deemed reliable. Cohen’s kappa is commonly used to determine the coefficient of agreement for categorical variables and ranges between –1 and 1.^28^ A score of 1 indicates perfect agreement, and a score of 0 indicates agreement no better than that expected by chance.^29^ A negative kappa would indicate agreement worse than that expected by chance.^30^ A Cohen’s kappa score of 0.6 was considered acceptable and 0.8 reflected a strong level of agreement.^31^ Incomplete GDMKQ forms were considered and analysed accordingly. Prior to conducting factor analysis, the suitability of performing factor analysis on this data set needed to be ascertained. For this reason, Bartlett’s test of sphericity and Kaiser–Meyer–Olkin (KMO), which measures sampling adequacy, were calculated.^32^ To be considered for factor analysis, Bartlett’s test of sphericity should be significant (*p* < 0.05). The KMO index ranges from 0 to 1, and the index can be interpreted as follows: 0.8 or above as meritorious; 0.7 or above as middling; 0.6 or above as mediocre; 0.5 or above as miserable; and below 0.5 as unacceptable.^32^

## Results

### Baseline characteristics of participants

A total of 124 participants with a mean age of 32.35 (s.d.: 4.96) years were included in this study. Two separate groups of 30 participants (*n* = 10 per language group) were enrolled to participate in the adaptation and pre-testing phases of the study (phase 1 and 2). Ninety-four participants (which included the 30 participants enrolled in phase 2) were enrolled in the reliability testing phase of the study. Median multigravida was 3 (IQR: 1) pregnancies in all three phases of this study (see Table 1).

**TABLE 1:** Baseline characteristics of participants per language group and phase of the study.

Variable	Overall (*n* = 30)						English (*n* = 10)						Afrikaans (*n* = 10)						isiXhosa (*n* = 10)						*p*
	Mean	s.d.	Frequency	%	Median	IQR	Mean	s.d.	Frequency	%	Median	IQR	Mean	s.d.	Frequency	%	Median	IQR	Mean	s.d.	Frequency	%	Median	IQR	
**Phase 1**																									
**Age (years)**	32.93	0.85	-	-	-	-	34.10	1.64	-	-	-	-	32.33	1.89	-	-	-	-	32.30	0.87	-	-	-	-	0.626
**Area**	-	-	-	-	-	-	-	-	-	-	-	-	-	-	-	-	-	-	-	-	-	-	-	-	0.021
- Cape Flats	-	-	15	50	-	-	-	-	4	40	-	-	-	-	2	20	-	-	-	-	9	90	-	-	-
- Cape Winelands	-	-	2	6.67	-	-	-	-	0	0	-	-	-	-	2	20	-	-	-	-	0	0	-	-	-
- Helderberg	-	-	3	10	-	-	-	-	1	10	-	-	-	-	2	20	-	-	-	-	0	0	-	-	-
- Northern-suburbs	-	-	10	33.33	-	-	-	-	5	50	-	-	-	-	4	40	-	-	-	-	1	10	-	-	-
**Gestational age (weeks)**	-	-	-	-	26	9	-	-	-	-	28.5	4	-	-	-	-	22	10	-	-	-	-	24	9	0.053
**Highest educational level**	-	-	-	-	-	-	-	-	-	-	-	-	-	-	-	-	-	-	-	-	-	-	-	-	0.251
- Grade 7–11	-	-	18	60	-	-	-	-	3	30.00	-	-	-	-	6	60.00	-	-	-	-	9	90.00	-	-	-
- Grade 12	-	-	8	26.67	-	-	-	-	5	50.00	-	-	-	-	2	20.00	-	-	-	-	1	10.00	-	-	-
- Tertiary Qualification	-	-	4	13.33	-	-	-	-	2	20.00	-	-	-	-	2	20.00	-	-	-	-	0	0	-	-	-
**Employment status**	-	-	-	-	-	-	-	-	-	-	-	-	-	-	-	-	-	-	-	-	-	-	-	-	0.122
- Employed	-	-	15	50	-	-	-	-	4	40.00	-	-	-	-	8	80.00	-	-	-	-	3	30.00	-	-	-
- Unemployed	-	-	15	50	-	-	-	-	6	60.00	-	-	-	-	2	20.00	-	-	-	-	7	70.00	-	-	-
**Gravida**	-	-	-	-	3	1	-	-	-	-	3	3	-	-	-	-	2.5	2	-	-	-	-	2.5	1	0.549
**Phase 2**																									
**Age (years)**	31.83	5.54	-	-	-	-	33	4.88	-	-		-	34	5.72	-	-	-	-	28.50	4.84	-	-	-	-	0.055
**Area**	-	-	-	-	-	-	-	-	-	-	-	-	-	-	-	-	-	-	-	-	-	-	-	-	0.201
- Cape Flats	-	-	13	43.33	-	-	-	-	2	20	-	-	-	-	4	40	-	-	-	-	7	70	-	-	-
- Cape Winelands	-	-	4	13.33	-	-	-	-	3	30	-	-	-	-	1	10	-	-	-	-	0	0	-	-	-
- Helderberg	-	-	3	10	-	-	-	-	2	20	-	-	-	-	1	10	-	-	-	-	0	0	-	-	-
- Northern-suburbs	-	-	10	33.33	-	-	-	-	3	30	-	-	-	-	4	40	-	-	-	-	3	30	-	-	-
**Gestational age (weeks)**	-	-	-	-	28.5	5	-	-	-	-	29	3	-	-	-	-	28.5	6	-	-	-	-	27	6	0.975
**Highest educational level**	-	-	-	-	-	-	-	-	-	-	-	-	-	-	-	-	-	-	-	-	-	-	-	-	0.170
- Grade 7–11	-	-	11	36.67	-	-	-	-	2	20	-	-	-	-	6	60	-	-	-	-	3	30	-	-	-
- Grade 12	-	-	15	50	-	-	-	-	7	70	-	-	-	-	3	30	-	-	-	-	5	50	-	-	-
- Tertiary qualification	-	-	4	13.33	-	-	-	-	1	10	-	-	-	-	1	10	-	-	-	-	2	20	-	-	-
**Employment Status**	-	-	-	-	-	-	-	-	-	-	-	-	-	-	-	-	-	-	-	-	-	-	-	-	0.439
- Employed	-	-	18	60	-	-	-	-	7	70	-	-	-	-	6	60	-	-	-	-	5	50	-	-	-
- Unemployed	-	-	12	40	-	-	-	-	3	30	-	-	-	-	4	40	-	-	-	-	5	50	-	-	-
**Gravida**	-	-	-	-	3	1	-	-	-	-	2.5	1	-	-	-	-	3	1	-	-	-	-	3	1	0.580
**Phase 3**																									
**Age (years)**	32.35	4.96	-	-	-	-	33.1	4.83	-	-	-	-	32.32	4.89	-	-	-	-	31.69	5.30	-	-	-	-	0.539
**Area**	-	-	-	-	-	-	-	-	-	-	-	-	-	-	-	-	-	-	-	-	-	-	-	-	0.049
- Cape Flats	-	-	39	41.93	-	-	-	-	7	23.33	-	-	-	-	12	38.71	-	-	-	-	20	62.50	-	-	-
- Cape Winelands	-	-	7	7.53	-	-	-	-	3	10	-	-	-	-	4	12.90	-	-	-	-	0	0	-	-	-
- Helderberg	-	-	13	13.98	-	-	-	-	5	16.67	-	-	-	-	5	16.13	-	-	-	-	3	9.38	-	-	-
Northern-suburbs	-	-	34	36.56	-	-	-	-	15	50	-	-	-	-	10	32.26	-	-	-	-	9	28.12	-	-	-
**Gestational age (weeks)**	-	-	-	-	28	5	-	-	-	-	29	6	-	-	-	-	28	6	-	-	-	-	28	5.5	0.799
**Highest educational level**	-	-	-	-	-	-	-	-	-	-	-	-	-	-	-	-	-	-	-	-	-	-	-	-	0.575
- Grade 7–11	-	-	33	35.48	-	-	-	-	9	30	-	-	-	-	11	35.48	-	-	-	-	13	40.62	-	-	-
- Grade 12	-	-	47	50.54	-	-	-	-	14	46.67	-	-	-	-	16	51.61	-	-	-	-	17	53.13	-	-	-
- Tertiary qualification	-	-	13	13.98	-	-	-	-	7	23.33	-	-	-	-	4	12.9	-	-	-	-	2	6.25	-	-	-
**Employment status**	-	-	-	-	-	-	-	-	-	-	-	-	-	-	-	-	-	-	-	-	-	-	-	-	0.202
- Employed	-	-	58	62.37	-	-	-	-	17	56.67	-	-	-	-	21	67.74	-	-	-	-	20	62.5	-	-	-
- Unemployed	-	-	35	37.63	-	-	-	-	13	43.33	-	-	-	-	10	32.26	-	-	-	-	12	37.5	-	-	-
**Gravida**	-	-	-	-	3	1	-	-	-	-	3	2	-	-	-	-	3	1	-	-	-	-	2	1	0.422

s.d., standard deviation; IQR, interquartile range.

#### Phase 1 and 2: Translation, cross-cultural adaptation, face and content validation

Forward and back translation of the GDMKQ to Afrikaans and isiXhosa was conducted by professional and independent freelance translators, and no issues with this process were indicated. All three versions of the new GDMKQ underwent four rounds of revision by an expert committee until a consensus was reached. The main themes identified were as follows: (1) simplified language, (2) inaccurate content, (3) proper linguistic and (4) cultural adaptation. To reduce guessing, the panellists also included ‘Please do not guess’ in the instructions. The original GDMKQ asked participants ‘The most common sign of hyperglycaemia (high blood sugar) is’ and listed three options ‘Sweating’, ‘Hunger’ and ‘Increased thirst’. The expert panel deemed this question as inappropriate and felt that there was no correct answer, and this question was modified. To ensure cross-cultural acceptability, linguistic changes (including grammar/spelling errors and semantic changes) as recommended by the panellists were implemented. Panellists also suggested changes to the options listed for questions focussing on the diet, as some food choices in the original questionnaire were not culturally appropriate in the Western Cape, South Africa. For changes, see Table 2.

**TABLE 2:** Summary of changes made to the Original Malaysian Gestational Diabetes Mellitus Knowledge Questionnaire.

Section	Changes
General	The original GDMKQ had 15 questions, unlike the SA-GDMKQ that only has 14 questions. The expert panel and participants agreed to remove the question that asked about their glucose levels. Throughout the questionnaire the word mellitus was removed and only included the term ‘gestational diabetes’.
Instruction	We added the following instruction to ensure participants who complete the questionnaire do not guess the answers. ‘Please do not guess. If you do not know which of the 3 statements is correct, choose the I DON’T KNOW option’.
Question 1	In the question replaced the word ‘occur’ with ‘happen’. The expert panel also felt the answers to the questions were confusing and made the answers clearer, example: ‘after pregnancy’ was changed to ‘directly after pregnancy’.
Question 2	The expert panel included the word ‘uncontrolled’ in the question as patients with GDM can have normal HGT levels. The answers were also simplified to ‘decreased/ increased’ instead of ‘lower/ higher than usual’.
Question 3	The question was simplified and condensed, and the expert panel also included pin-prick next to blood test to make it easier for patients to understand.
Question 4	The words ‘increased risk’ was used in the original questionnaire but changed to ‘higher chance’ to make it easier to understand.
Question 5	‘Increased chances’ was changed to ‘higher chances’ in the question.
Question 6	The question and answers were simplified.
Question 7	The original GDMKQ included food-groups like carbohydrates and fats, whereas the SA-GDMKQ included examples of the food-groups.
Question 8	All the answers were changes to food examples that is more common in SA.
Question 9	The original GDMKQ used rice as an example, the expert panel felt that white bread is a more common starch eaten in SA.
Question 10	The Original GDMKQ asked participants the most common sign; the SA-GDMKQ rather focused on a sign. The original GDMKQ listed hunger as a sign.
Question 11	Original GDMKQ questionnaire included medical terms which was simplified.
Question 12	One of the answers were changed completely. The original questionnaire included delivery time.
Question 13	The word ‘increased’ was replaced with ‘higher’ in the answers.
Question 14	The original GDMKQ included ‘is not very serious’ as an option. This was replaced with ‘only affects the baby’ in the SA-GDMKQ.

GDM, Gestational Diabetes Mellitus; GDMKQ, Gestational Diabetes Mellitus Knowledge Questionnaire; SA-GDMKQ, South African Gestational Diabetes Mellitus Knowledge Questionnaire; HGT, hematocrit.

All the participants who participated in the face and content validity phase felt that the questions were clear and easy to understand. Most participants ‘agreed’ (20%) and ‘strongly agreed’ (80%) that the SA-GDMKQ was easy to complete. All participants in the English and Afrikaans group deemed all the items important and applicable. However, one participant in the isiXhosa group felt that questions 11 and 12 were not important, ‘no one explained to me what normal sugar level is, they should tell me if it is high or not. I should not know this’. Overall, all participants agreed that the three versions of the SA-GDMKQ were well designed and well structured. The mean time taken to complete the questionnaires (all language groups) was 7.53 (s.d.: 1.36) min.

#### Phase 3: Internal consistency and test–retest reliability

**FIGURE 2:**
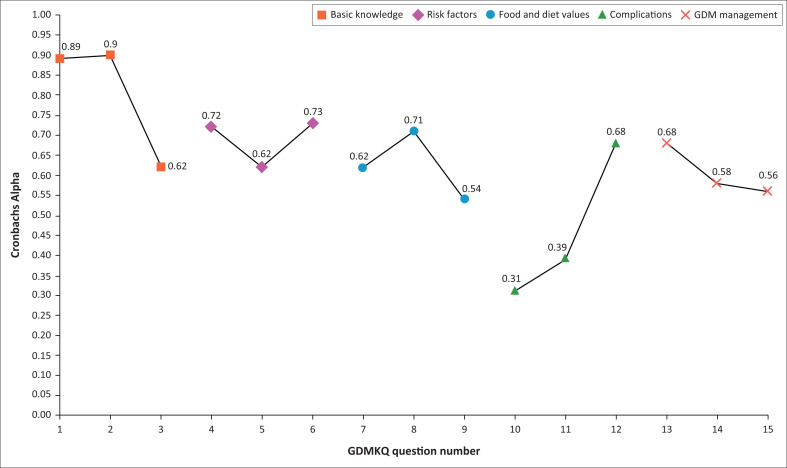
South African Gestational Diabetes Mellitus Knowledge Questionnaire Cronbach alpha results per question.

When testing the internal consistency of the three versions of the SA-GDMKQ, the Cronbach’s alpha values were as follows: α = 0.534 for the Afrikaans version, α = 0.434 for the English version and α = 0.621 for the isiXhosa version. Figure 2 illustrates the Cronbach’s alpha results per question for each questionnaire. Ninety-four participants agreed to complete the SA-GDMKQ, 2 weeks apart. One participant did not complete the second questionnaire, because of inability to attend the follow-up visit. The final sample for reliability assessment for each language group consisted of English-speaking (*n* = 30), Afrikaans-speaking (*n* = 31) and isiXhosa-speaking (*n* = 32) participants. On testing the test–retest reliability of the three versions of the GDMKQ, kappa (standard error [s.e.]) values ranged between −0.03 (0.18) and 0.89 (0.13) for the English version, between −0.07 (0.18) and 0.53 (0.13) for the Afrikaans version and between 0.28 (0.18) and 0.87 (0.17) for the isiXhosa version, respectively (Table 3). Question 1 pertaining to basic knowledge had the best agreement ranging between kappa (s.e.) 0.89 (0.13) and 0.53 (0.13) across the three versions of the SA-GDMKQ. Only one question, question 7, for food and diet values of the isiXhosa version, demonstrated strong agreement, kappa (s.e.) = 0.81 (0.13). All versions of the SA-GDMKQ had a statistically significant (*p* < 0.001) positive linear correlation between the total scores obtained in the first attempt, compared to the total scores obtained in the second attempt. The highest correlation was for the isiXhosa version (rho = 0.77, *p* < 0.001), followed by the Afrikaans version (rho = 0.73, *p* < 0.001) and lastly the English version (rho = 0.67, *p* < 0.001). On examination of the appropriateness of factor analysis, the KMO values were found to be less than 0.3 for all three versions of the SA-GDMKQ, and the Bartlett’s test of sphericity was non-significant (*p* > 0.05), indicating that the factor analysis processes were not appropriate.^31,32^ For this reason, exploratory factor analysis (EFA) was not conducted, and construct validity could not be assessed.

**TABLE 3:** Kappa for test-retest reliability per item of English, Afrikaans and isiXhosa versions of South African-Gestational Diabetes Mellitus Knowledge Questionnaire.

Topic	Question	English		Afrikaans		isiXhosa	
		Kappa	s.e.	Kappa	s.e.	Kappa	s.e.
Basic knowledge	1	0.89	0.13	0.53	0.13	0.87	0.17
	2	0.66	0.13	-	-	0.57	0.15
	3	0.60	0.12	0.33	0.13	0.42	0.10
Risk factors	4	−0.03	0.18	−0.07	0.18	0.56	0.11
	5	0.52	0.13	0.16	0.18	0.54	0.12
	6	0.00	0.00	0.00	0.00	0.49	0.11
Food and diet values	7	0.28	0.12	0.46	0.12	0.81	0.13
	8	0.54	0.13	0.37	0.12	0.55	0.12
	9	0.46	0.16	0.52	0.14	0.54	0.17
Management	10	0.32	0.11	0.33	0.11	0.33	0.12
	11	0.50	0.14	0.41	0.13	0.43	0.12
	12	0.51	0.13	0.43	0.12	0.59	0.12
Complication	13	0.19	0.18	0.31	0.17	0.64	0.13
	14	0.00	0.00	−0.05	0.16	0.51	0.17
	15	0.28	0.15	0.37	0.14	0.28	0.18

s.e., standard error.

## Discussion

This study found that all three versions had a good face and content validity, were applicable for the South African context, were easy to understand and were deemed feasible to assess the knowledge of pregnant women with GDM in the Western Cape of South Africa, although internal consistency and test–retest reliability require further investigation in larger sample sizes.

South Africa is a uniquely diverse country, consisting of multiple religions, cultures and traditions. Simultaneous translations in this study are comparable to previous South African research studies where multiple translations and simultaneous cross-cultural adaptation were conducted.^33^ This said, however, translation and cross-cultural adaptation are definitely more complex in a context like South Africa.^18,34,35^ The significant diversity, multiple national languages spoken and existence of various cultures and beliefs make it hard to conduct studies and to be inclusive. Typically, this complexity becomes a critical aspect of any research conducted in South Africa. With multiple translations and multiple cross-cultural adaptations, the cost of the research becomes significant, the sample size is influenced, and the research processes and outcomes are often affected. In addition, the processes are often compared to Western world research and not considered within the context. Many questionnaires and measures are developed in the less diverse Western world and in one language,^18,34,35^ and very little guidance exists regarding such translation, especially for the adaptation of a questionnaire from one English population to another.^18^ The processes followed in this study can therefore help inform logistics for larger future studies conducted in similar contexts and reduce initial layout costs. By considering the complexities in translating and adapting tools for such a diverse population, research in other diverse countries and contexts where situations can be challenging because of similar reasons can be informed.

The SA-GDMKQ demonstrated good face and content validity. With regard to face validity, all the participants felt that the SA-GDMKQ was clear and easy to understand and that all three versions were structured and designed in a way that made them easy to complete. In a context where a majority of the population are not well educated, access to healthcare is limited, capacity and time are limited for healthcare workers, etc., the ease of using a questionnaire is vital.^18,34^ The adapted versions of the SA-GDMKQ were not time-consuming and took a mean (s.d.) of 7.53 (1.36) min to complete. If the healthcare worker needs to assess a patient’s knowledge of GDM, they would be able to do so without taking too much time off their other daily tasks or from the patient’s consultation time. There was good agreement between the participant subgroups (per language) regarding the importance of including the various questions in the adapted versions of the SA-GDMKQ. The content validity of the three adapted versions of the SA-GDMKQ was therefore deemed appropriate. The three versions of the SA-GDMKQ could be recommended as practical questionnaires, especially in South Africa, where there are limited resources and staff, as well as capacity and time. Although further research is required, the results of the study provide preliminary evidence for the usefulness of such a questionnaire within the South African context.

The final versions of the SA-GDMKQ demonstrated low to moderate internal consistency; α = 0.534 for the Afrikaans version, α = 0.434 for the English version and α = 0.621 for the isiXhosa version (*n* = 94 participants). The internal consistency of the original GDMKQ was reported as α = 0.77, which is higher than reported in this study.^17^ According to our knowledge, the GDMKQ has not previously been translated or adapted, and this was the first attempt. The lower internal consistency values in this study could be because of the low numbers per language group and the low items as a whole for the questionnaire.^27^ Larger sample sizes in consequent studies are therefore encouraged to confirm or negate the findings in this study. However, this said, the lower internal consistency values should still be considered in the context of the type of questionnaire and the type of questions asked. Some questions were on symptoms, others were on nutrition and others related to the condition itself. For this reason, a lower internal consistency between questions could be expected because a respondent might have known more about one aspect of the condition than the other. In the current context, psychometric measures such as internal consistency should be viewed with caution, and all measures should be considered as a whole rather than be an absolute determinant for reliability testing.

All versions of the SA-GDMKQ demonstrated a statistically significant positive linear correlation between the total scores obtained in the first attempt, compared to the total scores obtained in the second attempt (*p* < 0.001). The correlation of the English version was (rho = 0.67, *p* < 0.001), the Afrikaans version was (rho = 0.73, *p* < 0.001) and the isiXhosa version was (rho = 0.77, *p* < 0.001). The Spearman’s correlation coefficient values, however, do not provide any insight into systematic errors that may be inherent in the measurement obtained during the completion of the SA-GDMKQ.^28^ Nevertheless, it did provide insights into the test–retest reliability of the adapted versions. Fluctuations of kappa scores of the SA-GDMKQ were seen between the different versions as well as between questions. This implies that the participants guessed the answers, even though the instructions to the participants were not to guess and choose the ‘I don’t know’ option. Furthermore, there were also kappa values less than 0. This raises concerns as it means that there is no agreement between the first and second attempts, indicating lower test–retest reliability. The kappa statistic considers the possibility of guessing, but the assumptions about the independence of the different attempts and other factors are not well supported, which lowers the estimate of agreement.^28^ Our interpretation of these findings may be that there could have been external or internal factors that influenced the participants’ responses on each of the two occasions. For example, participants did not receive their answers in the first round; therefore, they would not have known what they had correct. On the second attempt, they may have thought to choose another answer, in case the first one was incorrect, or they may have thought about the question during the 2-week period and either logically thought about it, asked their fellow patients, their families or the physicians if time allowed or may even have ‘Googled’ the answers, even though they were told not to. We, however, have no way of ascertaining if this was the case, and thus, for the purposes of the study, the methods of testing test–retest reliability were deemed adequate, although the results should be read with caution. Future studies should consider the possibilities of specific factors influencing test–retest reliability of similar questionnaires and how to mitigate against such influences.

This study had a few limitations that could be considered in future in similar research studies. Firstly, the exclusion of participants who displayed a ‘lack of comprehension’ could have possibly introduced bias. In the future, it is advised that researchers carefully consider how comprehension level is ascertained and how the exclusion of participants occurs. Secondly, it may be important to consider that different settings (such as the participant’s home or the clinic) may affect test–retest processes and results and should carefully be considered. Lastly, on preliminary examination, it was found that factor analysis was not appropriate as the sample size was inadequate. Future studies should therefore consider larger sample sizes, and factor analysis should be run to confirm the construct validity of the adapted questionnaire. However, as this study was a preliminary attempt, many of the logistical challenges associated with this type of research in such a unique population could be identified, and recommendations on how to improve upon these challenges when conducting research in similar contexts should be considered prior to broader testing. Therefore, although this study has limitations, it does provide a basis for future, larger studies on which the logistics of the research can be guided.

## Conclusion

In conclusion, this study found that the SA-GDMKQ version has a reasonable face and content validity, is applicable, is easy to use and understand, and is deemed feasible to assess the knowledge of women with GDM in the Western Cape area of South Africa. Further validation of the psychometric properties of the English, Afrikaans and isiXhosa versions of the SA-GDMKQ among larger sample groups is, however, warranted. It is envisaged that although this study was conducted in a small part of South Africa, the processes followed and challenges faced can be extrapolated to other parts of the country, and similar contexts across the globe, to inform research and policy when adapting outcome measures and questionnaires for specific populations. It is further recommended that in practice, the focus is directed to the accurate and appropriate assessment of knowledge among those living in rural and/or remote areas as accurate self-management education programmes in this population are vital.
